# Co-Sensitization to Silkworm Moth (*Bombyx mori*) and 9 Inhalant Allergens among Allergic Patients in Guangzhou, Southern China

**DOI:** 10.1371/journal.pone.0094776

**Published:** 2014-05-01

**Authors:** Baoqing Sun, Peiyan Zheng, Nili Wei, Huimin Huang, Guangqiao Zeng

**Affiliations:** State Key Laboratory of Respiratory Disease, National Clinical Center for Respiratory Diseases, Guangzhou Institute of Respiratory Diseases, First Affiliated Hospital, Guangzhou Medical University, Guangzhou, Guangdong Province, China; King’s College London, United Kingdom

## Abstract

**Objectives:**

This study aimed to investigate the profile of sensitization to silkworm moth (*Bombyx mori*) and other 9 common inhalant allergens among patients with allergic diseases in southern China.

**Methods:**

A total of 175 patients were tested for serum sIgE against silkworm moth in addition to combinations of other allergens: *Dermatophagoides pteronyssinus*, *Dermatophagoides farinae*, *Blomia tropicalis*, *Blattella germanica*, *Periplaneta americana*, cat dander, dog dander, *Aspergillus fumigatus* and *Artemisia vulgaris* by using the ImmunoCAP system. Correlation between sensitization to silkworm moth and to the other allergens was analyzed.

**Results:**

Of the 175 serum samples tested, 86 (49.14%) were positive for silkworm moth sIgE. With high concordance rates, these silkworm moth sensitized patients were concomitantly sensitized to *Dermatophagoides pteronyssinus* (94.34%), *Dermatophagoides farinae* (86.57%), *Blomia tropicalis* (93.33%), *Blattella germanica* (96.08%), and *Periplaneta americana* (79.41%). Moreover, there was a correlation in serum sIgE level between silkworm moth and *Dermatophagoides pteronyssinus* (r = 0.518), *Dermatophagoides farinae* (r = 0.702), *Blomia tropicalis* (r = 0.701), *Blattella germanica* (r = 0.878), and *Periplaneta americana* (r = 0.531) among patients co-sensitized to silkworm moth and each of these five allergens.

**Conclusion:**

In southern Chinese patients with allergic diseases, we showed a high prevalence of sensitization to silkworm moth, and a co-sensitization between silkworm moth and other five common inhalant allergens. Further serum inhibition studies are warranted to verify whether cross-reactivity exists among these allergens.

## Introduction

Recent studies in China and Western countries have indicated that airborne insect allergens play an equally important role as pollens and fungi in the incidence and development of allergic diseases [1]–[4]. Among various insects, silkworm moth (*Bombyx mori*) was identified as an important inhalant allergen in several studies worldwide [5]–[7]. In southern China where insects grow well in hot and humid climates, previous local epidemiological study in Guangzhou (unpublished data) showed that up to 40% of patients with respiratory allergy were sensitized to silkworm moth, and the prevalence of sensitization to silkworm moth was one of leading causes of allergy, immediately ranked after house dust mites and before cockroaches. This is interesting because silkworm moth is domesticated and is traditionally perceived as an occupational allergen found in silk-producing industries [8], [9]. In fact, silk has been described as a cause of asthma due to contact with bedcovers containing wild silk or silk waste [10]. Recent reports have found that silk waste from wild silk has become an important indoor allergen due to its use in a variety of other household products such as fillings in jackets, bed quilts and pillows [11]–[13]. Borelli and colleagues identified a patient who developed asthma while wearing a China made silk cardigan [14]. In a study by Wen CM et al, 64 children were found to carry silk-induced asthma with no occupational origin [15]. Therefore a high rate of sensitization to silkworm moth in a population beyond silk-workers should prompt a high probability of co-sensitization (defined as simultaneous sensitization to two or more allergens in a frequency higher than the chance expectation) or cross-reactivity among silkworm moth and other allergens. Unfortunately, there is little information available regarding silkworm moth-specific IgE level in Chinese patients and whether co-sensitization or cross reactivity exists between the silkworm moth and other common inhalant allergens. The present study was a retrospective evaluation of serum specific IgE (sIgE) against silkworm moth and 9 common inhalant allergens among patients from a large respiratory clinic in Guangzhou, southern China. We analyzed the pattern of correlation and the probability of co-sensitization or cross-reactivity between silkwom moth and these allergens.

## Materials and Methods

### Study Subjects

Between February 2010 and February 2013, among all patients who visited the Respiratory Clinic of First Affiliated Hospital of Guangzhou Medical University, the largest respiratory medical center in southern China, 5230 were referred for determination of serum specific IgE (sIg E) against common allergens in our laboratory for confirmatory diagnosis of allergic diseases. These subjects had been clinically confirmed to have allergic symptoms (including skin rashes, hives, red or itchy eyes, eczema, stuffy or runny nose, and sneezing), and were subsequently tested for different combinations of allergens, as decided individually by their attending physicians and indicated on a uniform order sheet. In total, the test for silkworm moth sIgE was ordered for 175 of these 5230 patients (81 males and 94 females), in addition to other allergen tests they received. These 175 patients fulfilled the following inclusion criteria: (1) a confirmed diagnosis of allergic asthma, allergic rhinitis, and/or chronic cough based on their clinical symptoms; and (2) a self-reported history of sneezing, eye itching, nasal congestion, runny nose or skin rash when exposed to dusts or insects. None of these subjects had: (1) known immunodeficiency, (2) clinically significant disorders of the lung, heart or liver, (3) a medical history or family history of chronic respiratory diseases, or (4) a history of respiratory infections or use of systemic corticosteroids during the previous 30 days. Based on medical record, all these 175 subjects came from the four large administrative districts of Guangzhou proper (Dongshan, Yuexiu, Haizhu and Baiyun Districts), thus representing a wide range of metropolitan coverage. The mean age of these patients was 45.42±18.93 years (range, 5–85 years). Table 1 shows the number of subjects tested for sIgE against silkworm moth and 9 other common inhalant allergens including *Dermatophagoides pteronyssinus* (Der p), *Dermatophagoides farinae* (Der f), *Blomia tropicalis*, *Blattella germanica*, *Periplaneta americana*, cat dander, dog dander, *Aspergillus fumigatus* and *Artemisia vulgaris*.

**Table 1:** The number of serum samples tested for both silkworm moth and 9 common inhalant allergens.

Tested allergens	Samples tested for both *Bombyx mori* and the allergen	*% of samples tested for both Bombyx more and the allergen among 175 subjects*
*Der p*	84	48%
*Der f*	152	87%
*Blomia tropicalis*	54	31%
*Blattella germanica*	77	44%
*Periplaneta americana*	37	21%
cat dander	103	59%
dog dander	101	58%
*Aspergillus fumigatus*	79	45%
*Artemisia vulgaris*	95	54%

The study protocol was approved by the Ethics Committee of First Affiliated Hospital, Guangzhou Medical University (Approval No.: GYFYY-2010-01-17). Written informed consent was obtained from all adults and from the parents or legal guardians of the children participating in this study.

### Specific IgE Measurement

Serum samples (5 ml from each of the 175 subjects) were tested for allergen-specific IgEs with the ImmunoCAP System (Themo Fisher, Clayton, NC, USA) according to the instructed procedures by the manufacturer. Based on the indication by the attending physicians, these 175 subjects were tested for sIgEs against silkworm moth and at least one of nine other allergens common in southern China, including *Der p*, *Der f, Blomia tropicalis, Blattella germanica, Periplaneta americana*, cat dander, dog dander, *Aspergillus fumigatus*, and *Artemisia vulgaris*. The sIgE level was calculated with a standard curve and expressed as concentration of kU/L. Based on the concentration, serum sIgE response to allergen was divided into 6 classes, namely class 0, <0.35 KU/L; class 1, ≥0.35 to <0.7 KU/L; class 2, ≥0.7 to <3.5 KU/L; class 3, ≥3.5 to <17.5 KU/L; class 4, ≥17.5 to <50 KU/L; class 5, ≥50 to <100 KU/L; and class 6, ≥100 KU/L. Responses with a sIgE level of ≥0.35 KU/L (class 1 or above) were defined as positive.

### Statistical Analysis

Data were analyzed with *SPSS* ver16.0 software package (SPSS Inc, Chicago, IL, USA). Chi-square test was used to determine the between-group differences of numerical data, and Spearman rank correlation analysis was used to test between-group correlations. A *P* value <0.05 was considered statistically significant.

## Results

### Sensitization to Silkworm Moth and 9 Common Inhalant Allergens

Of the 175 serum samples, 86 (49.14%) tested positive for silkworm moth-specific IgE. Based on the indication by attending physicians, they were also tested for sIgE against at least one of other 9 common inhalant allergens (Table 1). As shown in Table 2, the percentage of co-sensitization to silkworm moth were found in 59.52% of the subjects who tested positive for *Der p,* 38.16% for *Der f,* 51.85% for *Blomia tropicalis*, 63.64% for *Blattella germanica*, 72.97% for *Periplaneta americana*, 10.89% for dog dander, 7.77% for cat dander, 6.33% for *Aspergillus fumigatus*, and 4.21% for *Artemisia vulgaris*. These results can divide the sIgE response to silkworm moth and to the 9 common inhalant allergens into two concordance groups. The high concordance group, with a concordance rate ≥50%, comprised *Der p* (94.34%), *Der f* (86.57%), *Blomia tropicalis* (93.33%), *Blattella germanica* (96.08%), and *Periplaneta americana* (79.41%). The low concordance group which had a concordance rate <50% comprised cat dander (19.0%), dog dander (26%), *Aspergillus fumigatus* (16.13%), and *Artemisia vulgaris* (11.11%). A significant difference between these two groups was demonstrated by the chi-square test (*P*<0.001).

**Table 2:** Concordance in specific IgE detection results between silkworm moth and 9 common inhalant allergens.

Allergens	Co-sensitization to silkwormmoth (%)	Overall concordancerate (%)	Positive concordancerate (%)	Negative concordancerate (%)	Chi-square	Pvalue
Der p	59.52	86.9	94.34	74.19	1.45	0.23
Der f	38.16	84.21	86.57	82.35	1.04	0.31
Blomia tropicalis	51.85	88.89	93.33	83.33	0.17	0.68
Blattella germanica	63.64	93.51	96.08	88.46	0	1
Periplaneta americana	72.97	78.38	79.41	66.67	3.13	0.08
cat dander	7.77	66.02	19.05	98.36	29.26	<0.001
dog dander	10.89	68.32	26.19	98.31	26.27	<0.001
Aspergillus fumigatus	6.33	63.29	16.13	93.75	16.69	<0.001
Artemisia vulgaris	4.21	64.21	11.11	96.61	24.74	<0.001

### Serum sIgE Levels and Correlation Analysis on Sensitization to Silkworm and the High-concordance Group Allergens (with Concordance Rate ≥50%)

**Figure 1:**
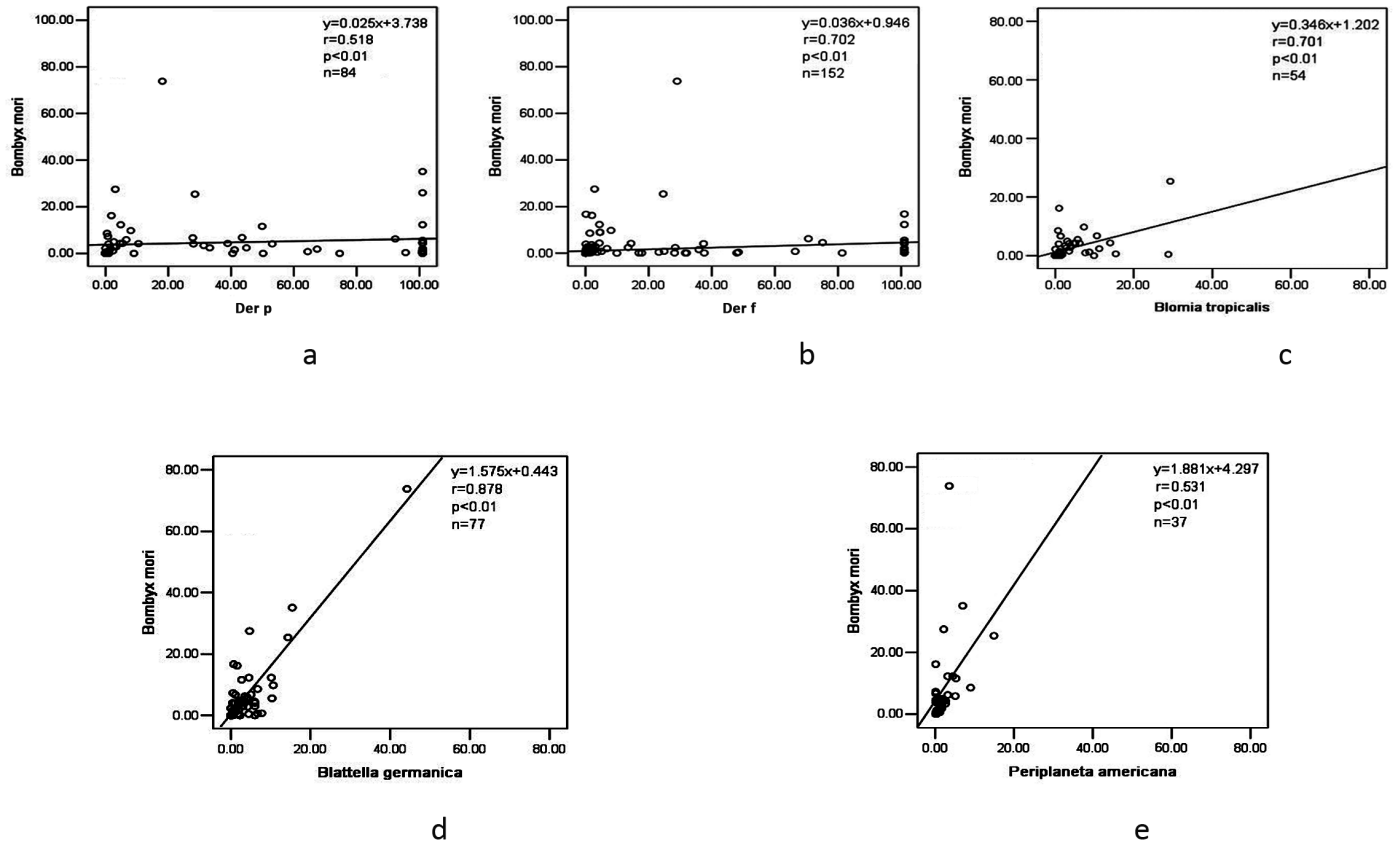
Spearman rank correlation in serum sIgE value between silkworm moth (*Bombyx mori*) and 5 common inhalant allergens: *Der p* (Figure 1a), *Der f* (Figure 1b), *Blomia tropicalis* (Figure 1c), *Blattella germanica* (Figure 1d), and *Periplaneta americana* (Figure 1e).

**Figure 2:**
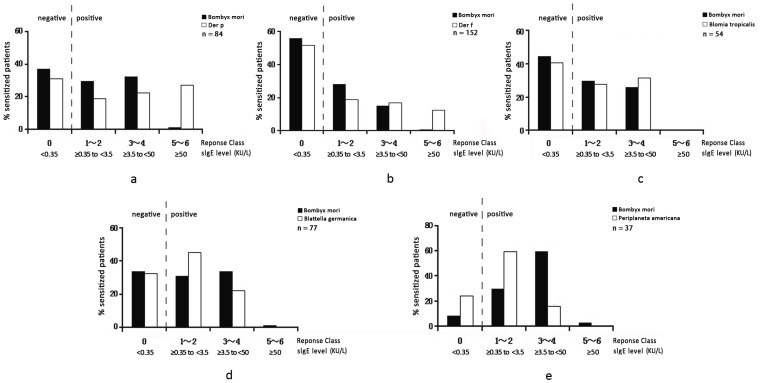
Percentage of patients sensitized to the silkworm moth (*Bombyx mori*) and 5 common inhalant allergens according to serum concentration of sIgE: *Der p* (Figure 2a); *Der f* (Figure 2b); *Blomia tropicalis* (Figure 2c); *Blattella germanica* (Figure 2d); *Periplaneta americana* (Figure 2e). The Y-axis shows the percentage of patients sensitized to allergens. The X-axis shows the concentration range of sIgE (class 1 through 6).

Figures 1a to 1e show the results of Spearman rank correlation analysis. The level of sIgE against silkworm moth was highly correlated with those against Der p (r = 0.518), Der f (r = 0.702), *Blomia tropicalis* (r = 0.701), *Blattella germanica* (r = 0.878) and *Periplaneta americana* (r = 0.531) (all P<0.01). Figures 2a to 2e show the percentages of patients sensitized to silkworm moth (*Bombyx mori*) and five common inhalant allergens according to serum sIgE level. For those who were co-sensitized to silkworm moth and mites, 27.4% of Der p and 12.5% of Der f positive patients had sIgE concentration ≥50 KU/L (classes 5 and 6) (Figures 2a, 2b); in contrast, only 1.19% and 0.66% of the silkworm moth sensitized patients had ≥50 KU/L sIgE for Der p and Der f, respectively. In patients co-sensitized to silkworm moth and *Blomia tropicalis*, none of them had sIgE level higher than 50 KU/L (Figure 2c).

As for cockroaches (Figures 2d and 2e), only 1.3% of *Blattella germanica*- and 2.7% of *Periplaneta americana*-positive patients co-sensitized to silkworm moth had ≥50 KU/L sIgE against moth.

Noticeably, in these five subgroups of high-concordance sensitization (Figure 2), the level of sIgE against silkworm moth was mostly below 50 KU/L; none of the patients sensitized to *Blomia tropicalis, Blattella germanica* or *Periplaneta americana* had sIgE >50 KU/L.

## Discussion

Cultivated silkworm (Bombyx mori) and wild silkworm (Antheraea spp) are the main sources of silk for manufacture of fabrics. During the production process, while silk threads go through several processes that can denature their allergenic proteins, silk waste is normally less carefully processed and retains antigenicity. In fact, it has been shown that a Chinese silk waste product intended for filling bed mattresses could contain several IgE-binding allergens in the range from 14 to 70 kD [16].

Unlike house dust mites, cockroaches, pet dander, molds, and pollens, silkworm moth is seldom reported in the literature with respect to its allergenicity and relationship with allergic diseases. Recently, sensitization to silkworm moth in southern China tends to be increasingly recognized in clinical practice, indicating a need to investigate whether the silkworm moth is also a common inhalant allergen. According to Li and colleagues, secretions, metabolites, setae, and scales of living moths, as well as the debris and lysates of dead moths, may be airborne triggers for allergic asthma and rhinitis [17]. In their study, 60 patients with asthma and 440 healthy forestry workers were skin prick tested with moth allergens, with the positive rates being 58.3% and 8.2% in the two groups, respectively [17]. When skin prick tests for a bigger panel of allergens were performed to further examine the allergic profile, they found that moth had the third highest positive rate after house dusts and house dust mites [18]. This observation is in accordance with our present finding that the positive rate of serum moth-specific IgE was 49.14% among allergic patients, suggesting that silkworm moth is an important inhalant insect allergen in Guangzhou area and the sensitization to silkworm moth may be closely related to allergic diseases, such as allergic asthma and allergic rhinitis.

Based on the correlation analysis, high concordance and strong correlation in positive sIgE reactivity was found between silkworm moth and other five common inhalant allergens (*Der p, Der f, Blomia tropicalis*, *Blattella germanica*, and *Periplaneta americana*), but not with another four inhalant allergens tested (cat dander, dog dander, *Aspergillus fumigatus*, and *Artemisia vulgaris*). The difference was statistically significant to show a co-sensitization pattern existing between silkworm moth and the five inhalant allergens from the high-concordance group among patients with allergic diseases in southern China. However, the current co-sensitization remains insufficient to demonstrate the presence of cross-reactivity between moth and these allergens.

Liu and colleagues has suggested a cross-reactivity between silkworm moth and cockroach and that arginine kinase may be the major cross-reactive antigenic component between the two insects [19]. Arginine kinase has also been confirmed to be responsible for the antigenic cross-reactivity among arthropods, such as shrimp [20], [21] and shellfish [22]. Although there were very few reports regarding whether a cross-reaction exists between silkworm moth and mites [23], [24], intra- and interspecies cross-reactivities among allergens from mites and cockroaches have been widely recognized. Twarog [25] showed the presence of cross-reactive antigens between *Periphlaneta americana* and *Blattella germanica*. Wu and colleagues demonstrated that the crude extracts of *Blattella germanica*, *Periplaneta americana*, *Blattella orientalis*, and *Blattella asahinai* contained common IgE-binding components [26]. A previous immunochemical study has reported cross-reactivity existing between house dust mites and snails, crustaceans, cockroaches, chironomids, and other mite allergens [27]. In the majority of related studies [7], [23], [28]–[30], house dust mite extracts proved to be a powerful inhibitor for the cross-inhibition tests, suggesting that it is the main source of many allergens resulting in hypersensitivity. The similar pattern was found in Chinese patients by Sun et al. [23] that 88% of the cockroach-positive patients were concurrently sensitized to *Der p.*

While many cross reactivity studies have been conducted on *Der p* and *Der f*, less information is available for *Blomia tropicalis*, one of the commonest mite species in tropical regions. Sporadic data have shown cross-reactivity between *Blomia tropicalis* and various mites, including *Der p*, *Lepidoglyphus destructor*, *Suidasia medanensis*, *Blomia kulagini*, and *Euroglyphus maynei* [29]–[33], as well as between *Ascaris* and tropomyosins from house dust mites and cockroaches [34]–[36].

In the present study, concurrent IgE sensitization was observed between silkworm moth and other 5 common inhalant allergens in allergic patients from southern China. Our findings provide serum sIgE data showing that silkworm moth is an important allergen for respiratory allergies in the region and a positive correlation exists between silkworm moth and the relevant mites and cockroaches. We acknowledge that owing to the retrospective nature of the study, we cannot conclude on the cross-reaction between these allergens. Further serum inhibition studies are warranted to verify such relationship, and if so, it would be worthwhile to find out whether it is attributed to arginine kinase.
